# First study of *in vitro* protective effect of *Lepidium meyenii* (Maca) on frozen–thawed bovine spermatozoa

**DOI:** 10.14202/vetworld.2022.1481-1488

**Published:** 2022-06-14

**Authors:** Johanna Leiva-Revilla, Miriam Rolón, Abolghasem Siyadatpanah, Maria de Lourdes Pereira, Veeranoot Nissapatorn

**Affiliations:** 1 Center for the Development of Scientific Research (CEDIC), Manduvira 635, CP 1255, Asunción, Paraguay; 2 Ferdows School of Paramedical and Health, Birjand University of Medical Sciences, Birjand, Iran; 3 CICECO-Aveiro Institute of Materials and Department of Medical Sciences, University of Aveiro, 3810-193 Aveiro, Portugal; 4 School of Allied Health Sciences, World Union for Herbal Drug Discovery, and Research Excellence Center for Innovation and Health Products, Walailak University, Nakhon Si Thammarat 80160, Thailand

**Keywords:** *Aberdeenangus*, antioxidant, bull, frozen-thawed semen, *Lepidium meyenii* (Maca), reproductive health

## Abstract

**Background and Aim:**

*Lepidium meyenii* Walp (Maca) is an herbaceous plant that grows in the Peruvian Andes and it has been widely used as a nutritional supplement and fertility enhancer and has been used in the treatment of a variety of diseases, such as rheumatism, respiratory disorders, and anemia. The most notable feature of Maca is its potent antioxidant capacity, which helps in the scavenging of free radicals and protection of cells from oxidative stress. This study aimed to evaluate the *in vitro* effect of Maca extract on thawed sperm cells from bulls.

**Materials and Methods:**

Three dilutions of 1, 10, and 100 mg/mL of Maca extract were incubated with frozen–thawed bovine semen and analyzed at 1, 3, and 24 h of exposure time, evaluating the activity of the extract on the DNA, motility, morphology, viability, integrity of the membrane and acrosome of spermatozoa.

**Results:**

The Maca extract improved the studied sperm parameters of motility, acrosome integrity, vitality, and DNA integrity of sperm cells at a concentration of 10 mg/mL, and at 1 mg/mL, an improvement was observed in the morphology and integrity of the membrane. However, the best activity of the Maca extract was observed on the DNA integrity of the sperm, which was effective at the three concentrations evaluated after 24 h of incubation.

**Conclusion:**

The results indicate that *L. meyenii* can help in maintaining spermatozoa cellular integrity after the frozen–thaw process, especially in the protection against DNA fragmentation. Therefore, Maca would be a feasible supplementation to protect sperm to maintain their fertile ability after thawing.

## Introduction

*Lepidium meyenii* Walp, commonly known as Maca, belonging to the Brassicaceae family and growing exclusively between 3500 and 4500 m above sea level, is an herb known as a traditional nutritional supplement, widely used in Peru, North America, and Europe, and is considered a millenary food for the Andean cultures [1]. According to numerous studies, the hypocotyls of Maca have been widely used as a nutritional supplement [1–4]. Furthermore, in folk medicine, it is used to increase fertility and sexual function, so it is popularly known as the “Andean Viagra” [4]. Maca has elements of high nutritional value, such as proteins, carbohydrates, essential amino acids, lipids, free fatty acids, and a series of secondary metabolites, such as macamides, alkaloids, and glucosinolates [4], and the composition and several medicinal effects of Peruvian Maca were reported very recently [2, 3]. Several studies have demonstrated the effects of Maca on semen quality, spermatogenesis, sperm count, and sperm motility in different species [1, 5–7]. These effects were observed in both healthy [1, 4, 6, 8] and animals with induced subfertility problems [9, 10]. In addition, a recent study has shown that Maca has a beneficial effect on the amount of fresh semen and its quality after storage at a temperature of 5°C for 72 h [6]. A systematic review based on trials on infertile and healthy men showed positive results of *L. meyenii* on semen quality [11]. A review of the properties of *L. meyenii* on sperm quality, sexual behavior, and male genital tract pathologies was recently published [12].

These data are interesting for the preservation of semen quality in reproductive biotechnology, such as artificial insemination (AI) and *in vitro* fertilization (IVF). In livestock, the use of AI has increased considerably in recent decades. From an industrial point of view, AI has several advantages over natural service, such as higher pregnancy rates and lower costs of transporting animals for riding [13]. A fundamental part of AI is the use of frozen semen. Therefore, samples must be carefully managed to prevent damage that occurs due to oxidative stress (OS) [6]. A strategy to prevent oxidative damage to sperm can be found in the supplementation of food with antioxidants or in the direct use of these supplements in conjunction with semen samples. One of the main characteristics of Maca is the presence of compounds such as macamides and glucosinolates that help in eliminating free radicals and protect cells from OS [14]. During spermatogenesis and steroidogenesis, sperm accumulate reactive oxygen species (ROS) [15]. For proper fertilization to occur, it is important to keep ROS levels low. In addition, sperm capacitation, hyperactivation, acrosome reaction, and sperm fusion require low levels of ROS to be more efficient [16]. ROS activity is a major concern for sperm quality both *in vivo* and during *in vitro* incubation, as well as during cold storage [17]. Tafuri *et al*. [17] observed on stallions that Maca contains some effective antioxidants, a high percentage of glucosinolates, and other important components with high antioxidant capacity that can protect sperm and keep ROS levels low. Despite the significance of sperm freezability affecting bull fertility and its economic impact on cattle farming, the efficiency of cryopreservation is still limited to 40–50% of cellular survival [18]. Although physical damage to membranes and biochemical changes has been linked to cryo-induced OS [19–21], the causes of differing freezabilities remain unknown.

This study aimed to analyze, for the first time, the *in vitro* effect of *L. meyenii* on frozen–thawed bull sperm to determine the activity of the extract as a protective agent against sperm cell damage after thawing.

## Materials and Methods

### Ethical approval

Not applicable.

### Study period and location

The study was carried out from March and December 2019 in the Assisted Reproduction Laboratory of the Center for the Development of Scientific Research (CEDIC), Asuncion, Paraguay.

### Experimental design

**Figure-1:**
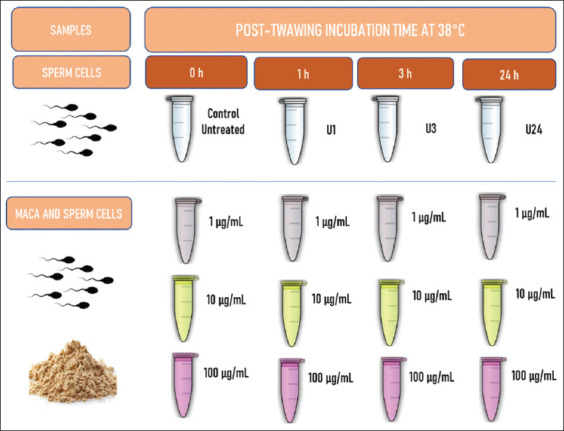
Design of the *in vitro* activity assays of *Lepidium meyenii* on frozen-thawed bull spermatozoa.

To evaluate the effect of *L. meyenii* extract on frozen–thawed sperm cells, three dilutions of the extract were tested at different times. Tests were conducted by preparing plastic tubes containing 1 mL of Sperm Prep Medium (SPM) (IVF Bioscience, Manhattan, USA) at a concentration of 2×10^6^ sperm/mL. After 1, 3, and 24 h of incubation at 38°C, control (U) and sperm cells with 1, 10, and 100 g/mL of Maca extract were evaluated (Figure-1). Samples were assessed for motility, morphology, membrane integrity, vitality, and DNA fragmentation at selected time points.

### Preparation of *L. meyenii* extracts

Commercial powder of *L. meyenii* Walp, composed of dry and raw yellow hypocotyl phenotypes [22, 23] (Maca en pó - Herbalsave Ltda., São Paulo, Brazil), was purchased in February 2019 in São Paulo, Brazil. A stock was prepared by weighing 1 mg of the powder and adding it to 1 mL of dimethyl sulfoxide (DMSO) (Sigma–Aldrich, St. Louis, USA) just before use. Three successive dilutions were prepared from the 1 mg/mL stock to be used at final concentrations of 1, 10, and 100 μg/mL in a final volume of 3 μL (DMSO <0.003%).

### Preparation of bull semen frozen–thawed samples

Two commercial straws of 0.25 mL of frozen bull semen (Aberdeen Angus, 18 months old) (Covepa group, Asunción, Paraguay) were thawed in a water bath at 37°C for 30 s. Both samples were pooled together and then washed twice with 2 mL of SPM medium by centrifugation at 1200 RPM for 5 min (Biosan LCM-3000, Riga, Latvia). The pellet was resuspended in 2 mL of SPM medium. Three independent experiments were performed.

### Sperm concentration

The number of sperm cells was counted by adding 10 μL of sperm suspension to a pre-warmed Makler chamber under an inverted phase-contrast microscope with a thermal stage (38°C) (IX70; Olympus, Centre Valley, PA, USA) at 200×. The final concentration to perform the routine and functional tests was 2×10^6^ sperm/mL in SPM medium.

### Motility assessment

Sperm motility and vigor were evaluated twice by the same observer after each treatment using an inverted phase-contrast microscope with a thermal stage at 38°C at 200× (IX70). A droplet (10 μL) of sperm was placed on a prewarmed microscope slide and covered to be counted immediately. The motility test was classified into four types: rectilinear, circular, static, and immobile tests. A minimum of 200 sperm cells were counted in each sample [24].

### Morphology test

For each group, 10 μL of sperm was placed on each slide for a thin smear and then air-dried for 5 min. Slides were stained with hematoxylin solution for 5 min, washed with distilled water, and air-dried for further processing. The presence of sperm cells were analyzed under a light microscope at 100× magnification (Axio LabA1, Zeiss, Germany) under oil immersion and classified as normal or abnormal conditions. Morphology of sperm cells refers to the structure of the individual sperm cells, and the minimum threshold for a bull to be classified as a satisfactory potential breeder is 70% normal, live cells [25]. Sperm defects (abnormalities) observed were grouped as follows: Head defects (e.g., detached head, defects in size and shape, nuclear vacuoles, and acrosomal defects), midpiece defects (e.g., distal midpiece reflex, bowed midpiece, and proximal droplet), and tail defects (e.g., bent tail and coiled tail) [26]. A total of 200 sperm cells were counted per slide from each group assayed.

### Membrane integrity − hypo-osmotic swelling (HOS) test

The procedure used for the HOS test was similar to the one described by Correa *et al*. [27]. The HOS test was performed by combining 0.1 mL of semen with 0.1 mL of hypo-osmotic solution prepared by mixing 7.35 g of sodium citrate 2H_2_O and 13.51 g of fructose in 1 L of distilled water. The solutions (sperm mixture) were then incubated at 37°C for 2 h. A total of 200 spermatozoa were counted in at least five different fields of view. Sperm swelling was assessed following incubation by placing a drop of a well-mixed sample on a slide. The slide was covered with a cover glass and observed under a phase-contrast microscope (IX70) at 400× magnification. The total proportion and different swelling patterns of swollen spermatozoa were calculated by dividing the number of reacted cells (100×) by the total spermatozoa counted in the same area. The proportion of swollen spermatozoa from a control sample was subtracted from the calculations. Swelling patterns were recorded and expressed on a percentage basis of the total spermatozoa that reacted to the HOS test.

### Acrosomal integrity and vitality test (AIT)

Trypan blue (Sigma–Aldrich, St. Louis, USA) and Giemsa dye (Merck, Darmstadt, Germany) stock solutions were prepared according to the manufacturer’s protocol. A total of 100 μL of each sample was placed in 100 μL of 2% Trypan blue and incubated for 10 min at 37°C. Samples were washed twice with 1 mL of SPM by centrifugation (6 min, 2000 RPM), and 10 μL of a smear was drawn and dried at 37°C. After that, slides were stained with 20% Giemsa for 40 min, rinsed underwater, and air-dried for further processing. The presence of sperm cells was analyzed under a light microscope (Axio LabA1, Zeiss, Germany) at 400× and classified into four groups: Dead with an acrosome, dead without an acrosome, alive with an acrosome, and alive without an acrosome. A minimum of 200 spermatozoa were counted in each group [28].

### DNA fragmentation with terminal deoxynucleotidyl transferase dUTP nick end labeling (TUNEL) assay

The amount of DNA fragmentation was evaluated by a TUNEL assay using a commercially available kit (*In situ* Cell Death Detection Kit, TMR red, Roche, Indianapolis, IN, USA), in which the free 3-OH ends of the DNA were labeled with fluorescein-conjugated dUTP by enzyme terminal deoxynucleotidyl transferase [29]. Briefly, fixed spermatozoa on the slides were washed three times for 5 min with phosphate-buffered saline (PBS) 1× and permeabilized with 0.1% (v/v) Triton X-100 containing 0.1% (w/v) sodium citrate for 2 min on ice. Samples were then incubated in 50 μL of the TUNEL reaction mixture for 1 h at 37°C in a dark and humidified atmosphere. For positive control, slides with spermatozoa were treated with RNase-free DNase I (400 U/mL, Qiagen, Valencia, CA, USA) at 23ºC for 10 min before incubation with the TUNEL reagent. For negative control, slides with spermatozoa were incubated with the TUNEL reagent in the absence of terminal deoxynucleotidyl transferase. Slides were washed three times with PBS, and mounted using a mounting medium (Vectasheld®, Vector Laboratories, Burlingame, CA, USA). Positive TUNEL staining was observed under a fluorescence microscope (Axio LabA1, Zeiss, Germany) using the filter set 14 Ex BP 510–560 nm excitation filter (Zeiss, Germany). The sperm TUNEL percentage was determined by counting the positive and negative stained spermatozoa. At least 200 cells were counted in each group.

### Statistical analysis

Values were compared using a one-way analysis of variance, followed by Dunnett’s *post hoc* test using the GraphPad Prism version 9.0 software (GraphPad Software, San Diego CA, USA). Differences were considered statistically significant at p < 0.05.

## Results

Table-1 details the effect of the treatment of the three concentrations of Maca dilutions at three exposure times on thawed and capacitated bovine sperm cells. The results of the motility, HOS, vitality, AIT, morphology, and TUNEL tests are expressed as the means of three experiments.

**Table 1:** Sperm quality evaluation tests of *Aberdeen Angus* treated with three concentrations of Maca and at three different times. The results are expressed as a percentage.

Test	Sperm cells status	Post-thawing incubation time and concentration of *L. meyenii*												

		0 h	1 h				3 h				24 h			
		Control	Control	1 µg/mL	10 µg/mL	100 µg/mL	Control	1 µg/mL	10 µg/mL	100 µg/mL	Control	1 µg/mL	10 µg/mL	100 µg/mL
Motility (%)	Rectilinear	42	50	46	63	57	54	54	55	53	49	60	53	48
	Circular	9	9	7	5	8	5	4	4	7	3	5	3	3
	Static	5	2	8	5	5	0	0	5	3	8	11	15	12
	Immotile	45	39	45	28	31	42	43	37	38	42	24	30	39
HOS (%)	Normal	44	46	44	59	47	44	52	47	47	44	42	46	41
	Abnormal	56	54	57	41	53	56	49	54	54	56	58	54	59
Acrosomal	Damaged - Died	35	19	26	27	27	19	37	21	29	30	37	34	43
Integrity (%)	Intact - Died	22	31	26	42	35	33	29	35	44	49	38	26	27
	Damaged - Live	7	2	1	2	3	15	3	2	2	1	1	1	0
	Intact - Live	37	48	48	30	36	34	32	43	26	21	25	40	30
Vitality (%)	Died	57	50	52	68	62	52	66	56	73	78	75	60	70
	Live	44	50	49	32	39	49	35	45	28	22	25	41	30
Morphology (%)	Normal	91	91	92	91	91	88	90	88	87	88	90	87	87
	Abnormal – Head	3	2	2	3	3	5	3	2	3	3	3	2	4
	Abnormal – Neck	3	3	3	3	3	4	3	5	6	6	5	4	5
	Abnormal - Tail	3	5	3	3	3	3	4	4	5	3	3	5	4
TUNEL (%)	Normal DNA	95.43	95.34	95.59	94.27	95.71	93.35	95.03	93.52	93.10	90.60	92.29	91.35	91.44
	Fragmented DNA	4.57	4.66	4.41	5.73	4.29	6.65	4.97	6.48	6.90	9.40	7.71	8.65	8.56

HOS=Hypo-osmotic swelling, TUNEL=DNA fragmentation with terminal deoxynucleotidyl transferase dUTP nick end labeling

**Figure-2:**
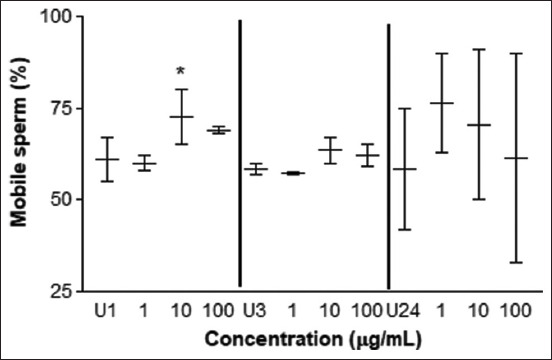
Motility test. Percentage of motile sperm cells treated with different concentrations of *Lepidium meyenii* versus control (U) at 1, 3, and 24 h of exposure. U1: untreated 1 h, U3: untreated 3h, U24: untreated 24 h of incubation time. The * means p < 0.05.

**Figure-3:**
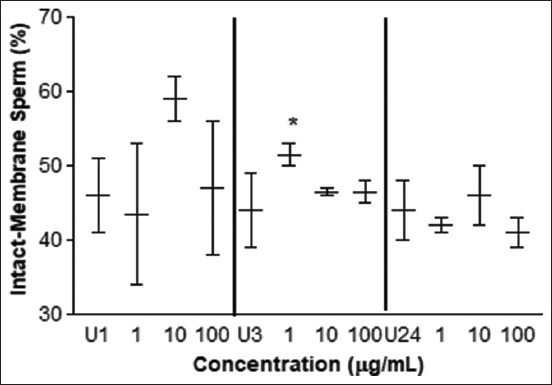
Hypo-osmotic swelling test. Percentage of sperm cells with intact plasma membrane treated with different concentrations of *L. meyenii* versus control (U) at 1, 3 and 24 h of exposure. U1: untreated 1 h, U3: untreated 3h, U24: untreated 24 h of incubation time. The *means p < 0.05.

The motility test determined an improvement with 10 μg/mL of *L. meyenii* at 1 h of incubation time but not in the other incubation times and concentrations assayed (Figure-2). Regarding the type of sperm motility, no differences in rectilinear, circular, or static movements were observed with treatments over time (p > 0.99) (Table-1). The HOS test showed a higher percentage of spermatozoa with greater plasma membrane integrity at 3 h of incubation when treated with 1 μg/mL of *L. meyenii* (Figure-3). No differences were observed in the other concentrations at 1, 3, and 24 h of incubation (p > 0.05).

**Figure-4:**
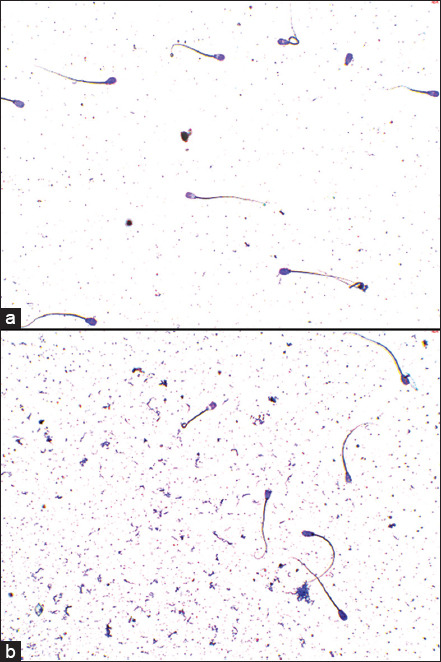
Acrosomal integrity and vitality test microscopic images at 3 h of incubation time under 400×. (a) Control sperm cells, (b) Sperm cells incubated with Maca at 100 μg/mL. Slightly stained acrosomes and/or cytoplasm indicates viable sperm, and strongly stained acrosomes and/or cytoplasm are damaged or dead sperm cells.

**Figure-5:**
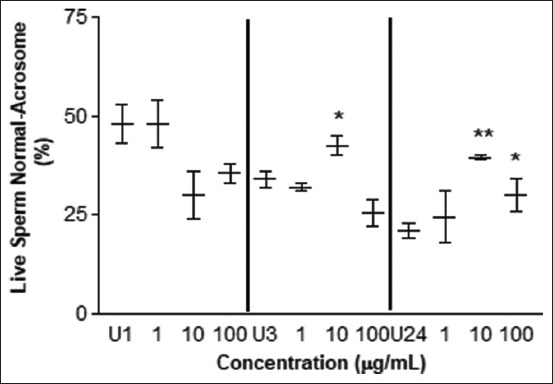
Acrosome intactness test. Percentage of intact acrosome in live sperm cells treated with different concentrations of *L. meyenii* at different times. U1: untreated 1 h, U3: untreated 3h, U24: untreated 24 h of incubation time. The * means p < 0.05 and ** p < 0.01.

In the AIT, no statistically significant differences were observed between the percentage of the integrity and damage of the acrosome in live and dead sperm cells (Table-1 and Figure-4). Nevertheless, an improvement in acrosome integrity was observed in live spermatozoa at 10 μg/mL after 3 h of exposure and at concentrations of 10 and 100 μg/mL after 24 h of incubation (Figure-5). Values below control were observed at concentrations of 10 μg/mL at 1 h of exposure (p < 0.01) and 100 μg/mL at 3 h of exposure (p < 0.01).

**Figure-6:**
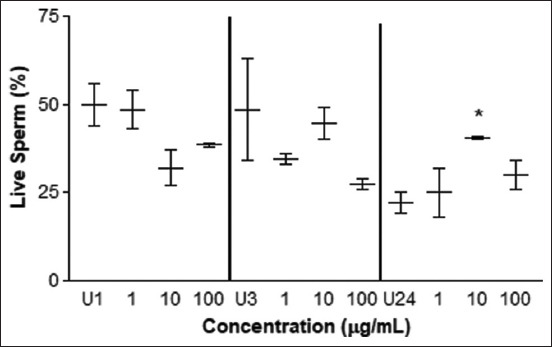
Vitality test. Percentage of live sperm cells treated with different concentrations of *L. meyenii* versus control (U). U1: untreated 1 h, U3: untreated 3h, U24: untreated 24 h of incubation time. The * means p<0.05.

**Figure-7:**
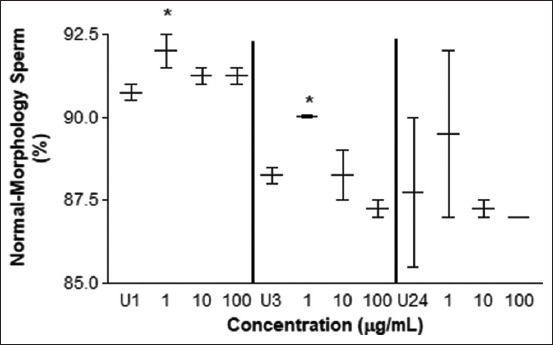
Morphology test. Percentage of sperm cells with normal morphology treated with different concentrations of *L. meyenii* versus control (U). U1: untreated 1 h, U3: untreated 3h, U24: untreated 24 h of incubation time. The ** means p<0.01.

The vitality test determined a greater amount of live spermatozoa treated with 10 μg/mL at 24 h of exposure (Figure-6), but values below the control were observed at concentrations of 10 and 100 μg/mL at 1 h of exposure (p < 0.05) and 100 μg/mL at 3 h of exposure (p < 0.05). The morphology test determined an improvement in sperm cells incubated with 1 μg/mL of *L. meyenii* after 1 and 3 h (p < 0.01) (Figure-7). No differences were observed at the other concentrations and incubation times (p > 0.05). The results obtained after 24 h of incubation were not statistically significant (p = 0.30). Values below control were observed at concentrations of 10 μg/mL after 3 h (p < 0.01) of exposure to 100 μg/mL (p < 0.05). By evaluating the morphological anomalies in the sperm head, neck, and tail, no differences were observed (Table-1) (p = 0.6, data not shown).

**Figure-8:**
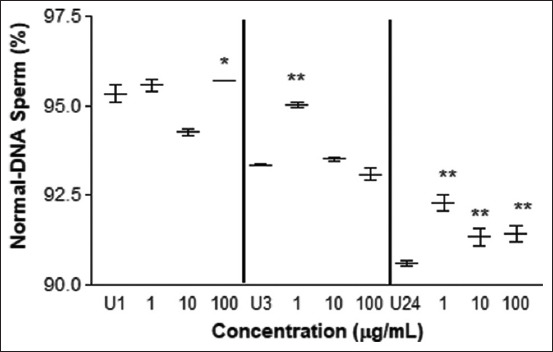
TUNEL test. Average percentage of sperm cells without DNA damage treated with different concentrations of *L. meyenii* versus control (U). U1: untreated 1 h, U3: untreated 3h, U24: untreated 24 h of incubation time. The * means p<0.05 and ** p<0.01.

In the TUNEL test, less sperm DNA damage was observed at concentrations of 100 μg/mL after 1 h of incubation, 1 μg/mL after 3 h, and 1, 10, and 100 μg/mL after 24 h of exposure (Figure-8). However, at concentrations of 10 μg/mL at 1 h of exposure (p < 0.01) and 100 μg/mL at 3 h of exposure (p < 0.05), values below the control were observed, indicating greater DNA damage.

## Discussion

This is the first study on the *in vitro* effect of Maca on bovine spermatozoa. The only *in vitro* study on Maca was conducted by Aoki *et al*. [7], and it was on mouse and human spermatozoa. The effects of the components of *L. meyenii* have been extensively studied in recent years. However, there are few studies performed on bulls [30, 31]. The present study showed the *in vitro* effects of *L. meyenii* on the sperm parameters of capacitated frozen–thawed bull semen. Several studies have shown that Maca has an effect on seminal quality in humans [5, 12, 32], mice [1, 2, 8–10], and stallions [6, 17] that is related to its antioxidant capacity [4, 11, 33–36] because, during cryopreservation, sperm is damaged by OS [18–21]. In addition to the high concentration of antioxidants, *L. meyenii* contains several secondary metabolites [3, 14, 22, 23], which help in keeping ROS levels low [14, 17]. A study showed that the secondary metabolites of Maca act on the ferric reducing antioxidant potential, hydroxyl radical scavenging ability, and 2,2-diphenyl-1-picrylhydrazyl free-radical scavenging capacity and increase the levels of superoxide dismutase and glutathione [37]. All of these secondary metabolites or a distinct combination of them may explain the potential effects of Maca on male fertility.

Regarding the tests carried out in this study, one of them is the HOS test, which evaluates whether an intact membrane is biochemically active [27]. Thus, the assessment of sperm membrane function appears to be a significant marker for the fertilizing capacity of spermatozoa [27]. The results obtained in the present study showed that frozen–thawed bovine spermatozoa reacted to HOS and that minimal swelling occurred at the lowest concentration evaluated. By itself, the HOS test is a much better predictor of the outcome of fertilization in humans than the sperm number, motility, or morphology parameters by themselves, as shown by the outcome of the zona-free hamster oocyte penetration assay and pregnancy rates in humans [27].

Regarding the morphology and motility test results, they improved at concentrations of 1 and 10 μg/mL, respectively, but an adverse effect on morphology was also observed at the highest concentration of 100 μg/mL. A study presented by Rubio *et al*. [38] indicated that male rats with acetate-induced damage on spermatogenesis treated with Maca showed an improvement in spermatogenesis, as well as increased motile sperm count and a count of sperm with normal morphology. The adverse effect of the concentration of 100 μg/mL was also observed in the TUNEL and AIT, which would indicate a hormetic effect of the extract in these parameters. Hormesis is defined as a cellular adaptive response to stressors that results in a biphasic dose–response relationship, such that low-dose stimulation results in a beneficial adaptation whereas a high dose results in a toxic effect [39]. Furthermore, this deleterious effect could be followed by an excessive concentration of antioxidants that reduce too many oxidants that, in low concentration, are necessary for spermatozoa functions. Therefore, the *in vitro* use of *L. meyenii* extract at high concentrations would not be recommended.

The best results of Maca on the DNA of spermatozoa were observed at 24 h of incubation at all concentrations tested. Maintaining the DNA integrity of thawed semen is critical in assisted reproduction because, in the case of bulls, sperm DNA fragmentation values of 7–10% were associated with low AI success [40], and genetic and epigenetic alterations in sperm can result in early pregnancy loss [41].

Sperm viability was significantly lower after 1 and 3 h of incubation with the Maca dilutions, except at 24 h. A previous study indicated that sperm viability is related to bull fertility [42]. However, as these are not the only parameters of importance in determining fertility, reliance on only these parameters will not intuitively provide enough information regarding potential fertility to be a useful predictor [43].

On spermatozoa, the plasma membrane, mitochondrial membrane, and acrosomal membrane contain polyunsaturated fatty acids, and hence, they are very susceptible to OS, especially during freezing procedures [44]. OS is one of the factors that increase cell damage due to ROS. Excessive ROS production in sperm is dangerous because of its negative effects on functional sperm count [45]. Acrosome integrity is one of the determinant factors for the success of fertilization. Only acrosome-intact spermatozoa can penetrate the zona pellucida and can fuse with the oocyte plasma membrane [46]. In this assay, no damage was observed in the acrosomal membrane of spermatozoa incubated with Maca at concentrations of 1 and 10 μg/mL after 3 and 24 h. This result showed the protective effect of the Maca components on the integrity of the spermatozoa membrane, which indicates that they might be acting as a protector agent.

## Conclusion

The *in vitro* results showed a general improvement in all sperm parameters analyzed (motility, vitality, acrosome integrity, morphology, and DNA fragmentation) in frozen–thawed bovine spermatozoa treated with *L. meyenii* extract, with the best results obtained at a concentration of 10 μg/mL. The observed improvement in these parameters plays an important role in predicting the sperm quality and thus the efficiency of embryo production. However, *in vivo* studies are required to elucidate the effects of *L. ­meyenii* in semen used to produce bovine embryos.

## Authors’ Contributions

JL, MR, VN: conception and designed the study, methodology, analysis and interpretation of data, investigation, writing-original draft. JL, MR, AS, MLP, VN: Methodology, investigation, analysis and interpretation of data.MR, VN: Project administration, investigation, writing-original draft, writing-reviewing and editing. All authors read and approved the final manuscript.

## References

[1] Gonzales G, Ruiz A, Gonzales C, Villegas L, Cordova A (2001). Effect of *Lepidium meyenii* (Maca) roots on spermatogenesis of male rats. Asian J. Androl.

[2] Wang S, Zhu F (2019). Chemical composition and health effects of Maca (*Lepidium meyenii*). Food Chem.

[3] da Silva Leitão Peres N, Cabrera Parra Bortoluzzi L, Medeiros Marques L.L, Formigoni M, Fuchs R.H.B, Droval A.A, Reitz Cardoso F.A (2020). Medicinal effects of Peruvian Maca (*Lepidium meyenii*):A review. Food Funct.

[4] Canales M, Aguilar J, Prada A, Marcelo A, Huamán C, Carbajal L (2000). Nutritional evaluation of *Lepidium meyenii* (MACA) in albino mice and their descendants. Arch. Latinoam Nutr.

[5] Gonzales G, Cordova A, Gonzales C, Chung A, Vega K, Villena A (2001). *Lepidium meyenii* (Maca) improved semen parameters in adult men. Asian J. Androl.

[6] Del Prete C, Tafuri S, Ciani F, Pasolini M.P, Ciotola F, Albarella S, Carotenuto D, Peretti V, Cocchia N (2018). Influences of dietary supplementation with *Lepidium meyenii* (Maca) on stallion sperm production and on preservation of sperm quality during storage at 5°C. Andrology.

[7] Aoki Y, Tsujimura A, Nagashima Y, Hiramatsu I, Uesaka Y, Nozaki T, Ogishima T, Shirai M, Shoyama Y, Tanaka H, Horie S (2019). Effect of *Lepidium meyenii* on *in vitro* fertilization via improvement in acrosome reaction and motility of mouse and human sperm. Reprod. Med. Biol.

[8] Inoue N, Farfan C, Gonzales G.F (2016). Effect of butanolic fraction of yellow and black Maca (*Lepidium meyenii*) on the sperm count of adult mice. Andrologia.

[9] Onaolapo A.Y, Oladipo B.P, Onaolapo O.J (2018). Cyclophosphamide-induced male subfertility in mice:An assessment of the potential benefits of Maca supplement. Andrologia.

[10] Valdivia Cuya M, Yarasca De La Vega K, Lévano Sánchez G, Vásquez Cavero J, Temoche García H, Torres Torres L, Cruz Ornetta V (2016). Effect of *Lepidium meyenii* (Maca) on testicular function of mice with chemically and physically induced subfertility. Andrologia.

[11] Lee M.S, Lee H.W, You S, Ha K.T (2016). The use of Maca (*Lepidium meyenii*) to improve semen quality:A systematic review. Maturitas.

[12] Tafuri S, Cocchia N, Vassetti A, Carotenuto D, Esposito L, Maruccio L, Avallone L, Ciani F (2019). *Lepidium meyenii* (Maca) in male reproduction. Nat. Prod. Res.

[13] Hasler J.F (2014). Forty years of embryo transfer in cattle:A review focusing on the journal Theriogenology, the growth of the industry in North America, and personal reminisces. Theriogenology.

[14] Long-Bo Z, Zhi-Lai Z, Qing-Xiu H, Min C, Li-Ping K (2019). Research progress on chemical constituents and bioactivities of *Lepidium meyenii*. Zhongguo Zhong Yao Za Zhi.

[15] Mathur P.P, D'Cruz S.C (2011). The effect of environmental contaminants on testicular function. Asian J. Androl.

[16] Bardaweel S.K, Gul M, Alzweiri M, Ishaqat A, ALSalamat H.A, Bashatwah R.M (2018). Reactive oxygen species:The dual role in physiological and pathological conditions of the human body. Eurasian J. Med.

[17] Tafuri S, Cocchia N, Carotenuto D, Vassetti A, Staropoli A, Mastellone V, Peretti V, Ciotola F, Albarella S, Del Prete C, Palumbo V, Esposito L, Vinale F, Ciani F (2019). Chemical analysis of *Lepidium meyenii* (Maca) and its effects on redox status and on reproductive biology in stallions. Molecules.

[18] Hitit M, Ugur M.R, Dinh T.T.N, Sajeev D, Kaya A, Topper E, Tan W, Memili E (2020). Cellular and functional physiopathology of bull sperm with altered sperm freezability. Front. Vet. Sci.

[19] Castro L.S, Hamilton T.R.S, Mendes C.M, Nichi M, Barnabe V.H, Visintin J.A, Assumpção M.E.O (2016). Sperm cryodamage occurs after rapid freezing phase:Flow cytometry approach and antioxidant enzymes activity at different stages of cryopreservation. J. Anim. Sci. Biotechnol.

[20] Gürler H, Malama E, Heppelmann M, Calisici O, Leiding C, Kastelic J.P, Bollwein H (2016). Effects of cryopreservation on sperm viability, synthesis of reactive oxygen species, and DNA damage of bovine sperm. Theriogenology.

[21] Peris-Frau P, Soler A.J, Iniesta-Cuerda M, Martín-Maestro A, Sánchez-Ajofrín I, Medina-Chávez D.A, Fernández-Santos M.R, García-Álvarez O, Maroto-Morales A, Montoro V, Garde J.J (2020). Sperm cryodamage in ruminants:Understanding the molecular changes induced by the cryopreservation process to optimize sperm quality. Int. J. Mol. Sci.

[22] Ganzera M, Zhao J, Muhammad I, Khan I.A (2002). Chemical profiling and standardization of *Lepidium meyenii* (Maca) by reversed-phase high-performance liquid chromatography. Chem. Pharm. Bull.

[23] Dini A, Migliuolo G, Rastrelli L, Saturnino P, Schettino O (1994). Chemical composition of *Lepidium meyenii*. Food Chem.

[24] Ax R.L, Dally M, Didion B.A, Lenz R.W, Love C.C, Varner D.D, Hafez B, Bellin M.E (2016). Semen evaluation. Reproduction in farm animals.

[25] Chenoweth P (2002). Bull breeding soundness exams and beyond.

[26] Menon A, Thundathil J, Wilde R, Kastelic J, Barkema H (2011). Validating the assessment of bull sperm morphology by veterinary practitioners. Can. Vet. J.

[27] Correa J.R, Zavos P.M (1994). The hypoosmotic swelling test:Its employment as an assay to evaluate the functional integrity of the frozen-thawed bovine sperm membrane. Theriogenology.

[28] Didion B.A, Dobrinsky J.R, Giles J.R, Graves C.N (1989). Staining procedure to detect viability and the true acrosome reaction in spermatozoa of various species. Gamete Res.

[29] Li X, Traganos F, Melamed M.R, Darzynkiewicz Z (1995). Single-step procedure for labeling DNA strand breaks with fluorescein-or bodipy-conjugated deoxynucleotides:Detection of apoptosis and bromodeoxyuridine incorporation. Cytometry.

[30] Clément C, Kneubühler J, Urwyler A, Witschi U.,, Kreuzer M (2010). Effect of Maca supplementation on bovine sperm quantity and quality followed over two spermatogenic cycles. Theriogenology.

[31] Clément C (2011). Relationship between colour type, environment and composition of the Andean plant Maca (*Lepidium meyenii* Walp.) and of its utility for enhancing fertility in breeding bulls.

[32] Melnikovova I, Tomas F, Huml L, Kolarova M, Lapcik O, Cusimamani E (2014). Effect of *Lepidium meyenii* on semen quality and reproductive hormones level in healthy adult men. Climacteric.

[33] Rodríguez-Huamán Á, Casimiro-Gonzales S, Chávez-Pérez J.A, Gonzales-Arimborgo C, Cisneros-Fernández R, Aguilar-Mendoza L.Á, Gonzales G.F (2017). Antioxidant and neuroprotector effect of *Lepidium meyenii* (Maca) methanol leaf extract against 6-hydroxy dopamine (6-OHDA)-induced toxicity in PC12 cells. Toxicol. Mech. Methods.

[34] Korkmaz S (2018). Anti-oxidants in Maca (*Lepidium meyenii*) as a supplement in nutrition. Anti-oxidants in foods and its applications.

[35] Lee Y.K, Chang Y.H (2019). Physicochemical and anti-oxidant properties of methanol extract from Maca (*Lepidium meyenii* Walp.) leaves and roots. Food Sci. Technol.

[36] Buyanbadrakh E, Hong H.S, Lee K.W, Huang W.Y, Oh J.H (2020). Anti-oxidant activity, macamide B content and muscle cell protection of Maca (*Lepidium meyenii*) extracted using ultrasonication-assisted extraction. Microbiol. Biotechnol. Lett.

[37] Gan J, Feng Y, He Z, Li X, Zhang H (2017). Correlations between anti-oxidant activity and alkaloids and phenols of Maca (*Lepidium meyenii*). J. Food Qual.

[38] Rubio J, Riqueros M.I, Gasco M, Yucra S, Miranda S, Gonzales G.F (2006). *Lepidium meyenii* (Maca) reversed the lead acetate induced-damage on reproductive function in male rats. Food Chem. Toxicol.

[39] Calabrese E.J, Mattson M.P, Calabrese V (2010). Resveratrol commonly displays hormesis:Occurrence and biomedical significance. Hum. Exp. Toxicol.

[40] Karoui S, Díaz C, González-Marín C, Amenabar M.E, Serrano M, Ugarte E, Gosálvez J, Roy R, López-Fernández C, Carabaño M.J (2012). Is sperm DNA fragmentation a good marker for field AI bull fertility?. J. Anim. Sci.

[41] Khadem N, Poorhoseyni A, Jalali M, Akbary A, Heydari S.T (2014). Sperm DNA fragmentation in couples with unexplained recurrent spontaneous abortions. Andrologia.

[42] Rodríguez-Martínez H (2013). Semen evaluation techniques and their relationship with fertility. Anim. Reprod.

[43] Kumaresan A, Johannisson A, Al-Essawe E.M, Morrell J.M (2017). Sperm viability, reactive oxygen species, and DNA fragmentation index combined can discriminate between above-and below-average fertility bulls. J. Dairy Sci.

[44] Chelucci S, Pasciu V, Succu S, Addis D, Leoni G.G, Manca M.E, Naitana S, Berlinguer F (2015). Soybean lecithin-based extender preserves spermatozoa membrane integrity and fertilizing potential during goat semen cryopreservation. Theriogenology.

[45] Rath D, Bathgate R, Rodriguez-Martinez H, Roca J, Strzezek J, Waberski D (2009). Recent advances in boar semen cryopreservation. Soc. Reprod. Fertil.

[46] Celeghini E.C.C, Nascimento J, Raphael C.F, Andrade A.F.C, Arruda R.P (2010). Simultaneous assessment of plasmatic, acrosomal, and mitochondrial membranes in ram sperm by fluorescent probes. Arq. Bras. Med. Vet. Zootec.

